# Possible mechanism and Atorvastatin-based treatment in cupping therapy-related subdural hematoma: A case report and literature review

**DOI:** 10.3389/fneur.2022.900145

**Published:** 2022-07-22

**Authors:** Tangtang Xiang, Xinjie Zhang, Yingsheng Wei, Dongyi Feng, Zhitao Gong, Xuanhui Liu, Jiangyuan Yuan, Weiwei Jiang, Meng Nie, Yibing Fan, Yupeng Chen, Jiancheng Feng, Shiying Dong, Chuang Gao, Jinhao Huang, Rongcai Jiang

**Affiliations:** ^1^Department of Neurosurgery, Tianjin Medical University General Hospital, Tianjin, China; ^2^Key Laboratory of Post Neuro-injury Neuro-repair and Regeneration in Central Nervous System, Ministry of Education and Tianjin, Tianjin, China

**Keywords:** cupping therapy, Atorvastatin, dexamethasone, subdural hematoma (SDH), Intracranial Pressure Reduction

## Abstract

Subdural hematoma (SDH) is one of the most lethal types of traumatic brain injury. SDH caused by Intracranial Pressure Reduction (ICPR) is rare, and the mechanism remains unclear. Here, we report three cases of SDH that occurred after substandard cupping therapy and are conjected to be associated with ICPR. All of them had undergone cupping treatments. On the last cupping procedure, they experienced a severe headache after the cup placed on the occipital-neck junction (ONJ) was suddenly removed and were diagnosed with SDH the next day. In standard cupping therapy, the cups are not usually placed on the ONJ. We speculate that removing these cups on the soft tissue over the cisterna magna repeatedly created localized negative pressure, caused temporary but repeated ICPR, and eventually led to SDH development. The Monro-Kellie Doctrine can explain the mechanism behind this - it states that the intracranial pressure is regulated by a fixed system, with any change in one component causing a compensatory change in the other. The repeated ICPR promoted brain displacement, tearing of the bridging veins, and development of SDH. The literature was reviewed to illustrate the common etiologies and therapies of secondary ICPR-associated SDH. Despite the popularity of cupping therapy, its side effects are rarely mentioned. This case is reported to remind professional technicians to fully assess a patient's condition before cupping therapy and ensure that the cups are not placed at the ONJ.

## Introduction

Intracranial Pressure Reduction (ICPR) comprises decreased cerebrospinal fluid (CSF) volume and pressure with persistent orthostatic headache as the primary symptom and usually results from CSF leakage (1). Although ICPR is usually a temporary condition, it may progress and increase the risk of developing a rare and potentially fatal complication—ICPR -associated intracranial subdural hematoma (SDH) (1, 2). The mechanism behind this can be explained by the Monro-Kellie Doctrine. It states that the intracranial pressure is regulated by a fixed system, with any change in one component causing a compensatory change in the other (3, 4). Therefore, when the intracranial pressure drops, the subdural space expands, causing a caudal descent of the brain. A sudden shift of the brain may cause traction at the arachnoid mater and venous structures on the brain's surface, followed by the development of unilateral or bilateral SDH due to the tearing of these veins in the subdural space (4, 5) as the venous wall is small and weak and thus may be the predisposed location for rupture (5).

Cupping treatment, a traditional medicine practiced for approximately 3,000 years in which local suction is created on the skin by applying heated cups, has become a popular alternative approach for treating various ailments worldwide, including spinal pain and herpes neuralgia (6, 7). Some studies have shown its effects on both local and system, wherein cupping stretches the skin and underlying tissue and dilates the capillaries, eventually leading to capillary rupture and ecchymosis (8). Macrophages phagocytose the leaking hemoglobin and stimulate the production of Heme oxygenase-1 to metabolize these hemoglobins, and this enzyme catalyzes the hemoglobin heme into biliverdin, carbon monoxide, and iron (9). These substances have been shown to have antioxidant and anti-inflammatory effects and stimulate a shift of macrophages to the anti-inflammatory M2 phenotype (10–13). However, cupping treatment side effects and operational considerations are rarely mentioned in the literature.

**Figure 1:**
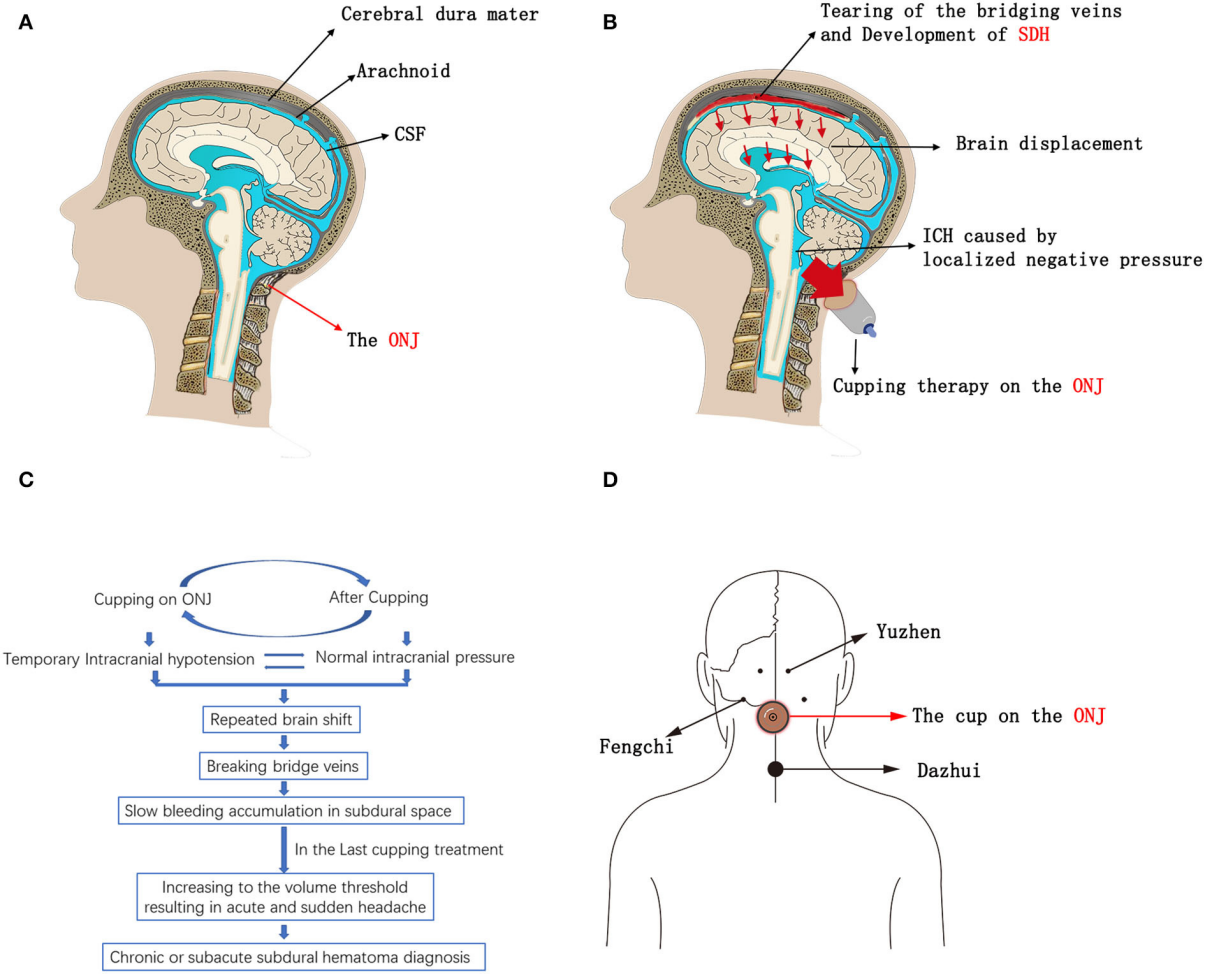
**(A–C)** Schematics of SDH development due to the substandard Cupping therapy on the ONJ. **(D)** Schematics of the most common anatomical sites that are close to the ONJ in traditional Chinese cupping therapy; Yuzhen (near the external occipital protuberance), Fengchi (near the base of the occiput), and Dazhui (near the seventh cervical vertebra). CSF, Cerebrospinal Fluid; ONJ, Occipital-neck Junction; SDH, Subdural Hematoma; ICPR, Intracranial Pressure Reduction.

Here, we identified three patients who developed SDH after receiving substandard Chinese cupping therapy. All three patients had no or only mild neurological symptoms before the cupping treatment, but they developed SDH after subsequent cupping treatment, during which the cup was applied on the occipital-neck junction (ONJ). In standard cupping therapy, the cups are usually placed on the area comprising muscle, vessels, and nerves and not on the ONJ (14–16). According to the literature, the negative pressure caused by cupping therapy may be as high as −225 and −375 mmHg (15). The tissue on the ONJ is typically soft, connected to the cisterna magna, and susceptible to physical effects. Therefore, we speculate that the localized negative pressure on the soft tissue over the cisterna magna caused instant secondary ICPR. According to the Monro-Kellie Doctrine, instant ICPR promotes brain displacement, bridging vein tear, and SDH development (Figures 1A–C). The brain shift should become more severe if the intracranial pressure switches between low and normal multiple times. In traditional Chinese medicine, the most common anatomical sites of cupping therapy, including Yuzhen (near the external occipital protuberance), Fengchi (near the base of the occiput), and Dazhui (near the seventh cervical vertebra), are close to the ONJ, which may be the cause of this misoperation (Figure 1D).

This is the first report to elucidate the potential risk of this traditional cupping therapy for developing SDH if the cups are wrongly positioned. All patients, except one, were successfully treated with conservative Atorvastatin-based therapy (ATO). Moreover, we have reviewed the literature on the etiologies and therapies of secondary ICPR-associated SDH.

## Case description

### Case 1

A 54-year-old man was admitted to our hospital complaining of a severe headache one day after his 10th cupping procedure. Before that, He was administered cupping therapy once a week for approximately 3 months because of lumps on his neck. One of the cups was positioned on the ONJ. He clearly remembered that he experienced a sudden severe headache on the 10th cupping procedure when the cup placed on the ONJ was suddenly removed. He had no history of trauma but had a hypertension history, controlled with telmisartan.

**Figure 2:**
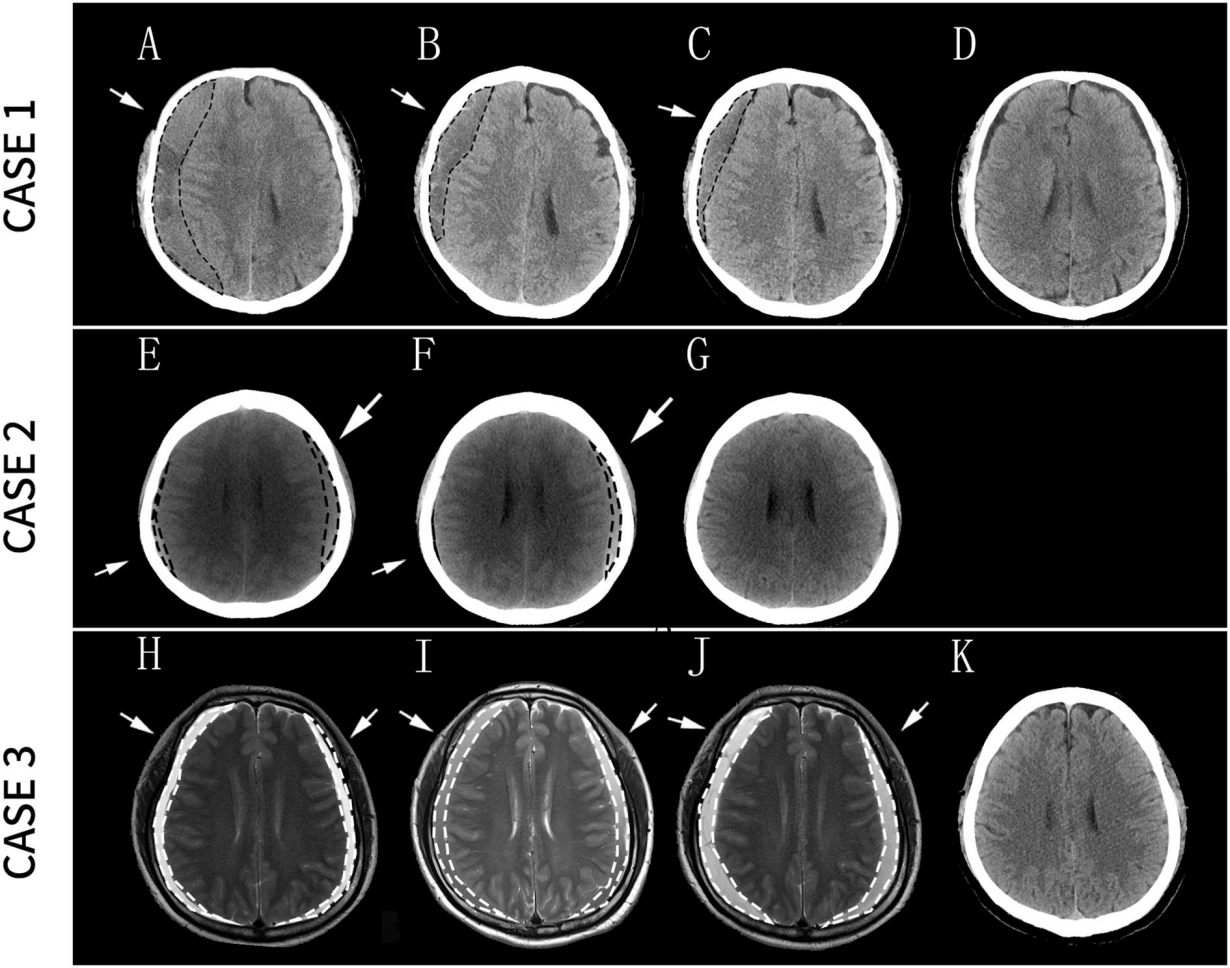
Case 1: **(A)** Diagnosis of right SDH; **(B)** The size of SDH significantly decreased after 9 days of the treatment; **(C)** SDH continued to decrease in size after 1 month of treatment; **(D)** the residual hematomas resolved 3 months after the treatment. Case 2: **(E)** Diagnosis of bilateral SDH; **(F)** the size of the right SDH significantly decreased after 1 month of the treatment; **(G)** the residual hematomas resolved 4 months after the treatment. Case 3: **(H)** Diagnosis of bilateral SDH; **(I)** Headache considerably decreased after 15 days of treatment but without a reduction in the size of the hematoma on MRI images; **(J)** Seventeen days later, a head MRI revealed an expansion of the SDH, and the patient underwent surgery after 2 days; **(K)** Two months later, the patient recovered well without any neurological sequelae. Arrows and dashed lines indicate the locations of SDH. SDH, subdural hematoma; MRI, magnetic resonance imaging.

After admission, potential brain diseases were excluded by the head MRA. CT of the head revealed a large SDH on the right side (Figure 2A). He walked to visit our clinic with a mild motor deficit (left muscle strength test, 3/5; right muscle strength test, 4/5). Blood examination results, including platelet count, PT, and activated partial thromboplastin time, were normal. He was not administered anticoagulation or antiplatelet therapy.

Considering the mild symptoms and his reluctance to undergo surgery, he was treated with a drug regimen comprising daily 20 mg of Atorvastatin combined with dexamethasone (DEX) with stepwise-decreasing dosing (2.25 mg daily for the first week, 1.5 mg daily for the next 2 weeks, and 0.75 mg daily for the last week) for a total of 4 weeks based on the modified protocol that we utilized previously (17). After 9 days of the treatment, the hematoma size was significantly reduced (Figure 2B), and the patient's symptoms were relieved. After 1 month of the treatment, the hematoma continued to decrease in size (Figure 2C). Three months after the treatment, the residual hematomas and all the neurological symptoms resolved (Figure 2D), and the patient was symptom-free. No side effects had been observed during the treatment.

### Case 2

A 48-year-old woman visited our outpatient clinic complaining of persistent headache, nausea, and vomiting on the day after her fourth cupping procedure. She had an injury to the head a month prior and then experienced occipital discomfort. Furthermore, she received traditional Chinese cervical massage, acupuncture, and cupping therapy once a week. The discomfort was relieved slightly after these treatments. However, during the 4th cupping procedure, she suddenly had a severe headache when the cup was removed from the ONJ.

C.T. of the head revealed bilateral SDH (Figure 2E). The neurological tests were regular, as were the results of laboratory tests. No other treatment, such as antithrombotic or antiplatelet medications, was prescribed at that time. She was later treated with an ATO at the previously mentioned dose. One month later, she was re-examined at our outpatient service, showing significantly relieving symptoms and a reduction in the size of the right hematoma (Figure 2F). After 4 months of the combined treatment, CT showed that the residual hematomas resolved (Figure 2G). The patient had no complaints during the follow-up period.

### Case 3

A 44-year-old man with a five-year history of hypertension and gout was admitted to our hospital 1 day after his fourth cupping procedure. He was hit on the head by a heavy object a month previously. Ten days after the accident, he developed a mild headache and neck muscle spasms. He underwent traditional medical treatment, including cupping and acupuncture, once a week. One of the cups was positioned on the ONJ. The patient's neck discomfort was successfully alleviated after the treatment. However, after the third round of cupping therapy, his headache worsened, although it could be relieved by lying down. Furthermore, he clearly remembered an aggravating and constant headache with dizziness and vomiting after the cup on his ONJ was removed during the 4th cupping procedure.

An MRI of the head was conducted and showed bilateral SDH (Figure 2H), with slight delirium, but without other marked neurological impairment. The patient had not received coagulation-related medications prior to the onset of the headache. His PT, bleeding time and clotting time were within normal ranges. As mentioned previously, he rejected surgical therapy and was treated with the ATO. After 15 days of the treatment, although there was no reduction in the hematoma size on MRI images (Figure 2I), his headache considerably decreased, and he was discharged from the hospital with continuous oral administration of Atorvastatin and DEX. Seventeen days later, his headache had exacerbated. Head MRI revealed a slight expansion of the bilateral frontotemporal paroccipital chronic SDH (Figure 2J). A new round of combination therapy was restarted at the same dose with intensive monitoring of the patient's vital signs. However, 2 days later, his symptoms worsened, and the hematoma was surgically drained. Two months later, the patient recovered well without any neurological sequelae (Figure 2K).

## Discussion

This study presents three patients who had no or only mild neurological symptoms before the cupping treatment but were diagnosed with SDH after placing the cup on the ONJ. According to the previous literature describing the image features of subdural hematoma, the imaging signal strength of the hematoma is closely related to its types (18–21). Acute subdural hematoma normally features hyperdense in CT images and hyper signal in MRI T1 and T2. In this report, the signal of the hematomas of the first patient is low with some trabecular in CT images. The 2nd case has iso-density hematomas in the CT image. The CT image is not obtained in the 3rd case, while his hematomas display a mixed high and low intensity in T2. Thus, we tend to think of their hematoma as chronic subdural or subacute hematoma. In addition, according to a study by Nakaguchi et al., chronic subdural hematoma (CSDH) has a natural history. It develops initially as the.homogeneous type sometimes progresses to the laminar type, matures in the separated stage, and eventually passes through the trabecular stage during absorption (19). In the first case, hematoma developed trabeculae with a moderately high density lying in a low-density to an iso-dense matrix, which suggested that it had been developing for a long time. The patient underwent 10 cupping treatments and had a long course of illness, consistent with his imaging findings.

How cupping therapy leads to SDH is a key issue in this study. The chronic and subacute hematoma indicated that the patients had SDH prior to the last cupping treatment. All three patients had multiple cupping experiences, and the second and third patients had a history of head trauma. Thus, it is reasonable to speculate that these patients developed SDH due to the early cupping therapy or head trauma, and they had an acute outbreak headache because the volume increased to the threshold size after the last cupping. The literature has described that the ICPR caused by spinal CSF leakage can lead to delayed neurological deterioration in a patient with SDH (22).

Noticeably, When the patient is cupping at the ONJ, ICPR occurs when the cups create a localized negative pressure and reach the maximum negative value when the cup is suddenly removed. Then, the ICP may gradually return to normal (or higher) without external intervention. This may explain why we did not find more specific symptoms of ICPR in these patients after hospitalization or in their history.

We also reviewed the literature on patients with SDH with secondary ICPR (Table 1). Although the risk of neurological complications following spinal anesthesia was evaluated to be only 1/20,000 to 1/30,000 (46), and to be only 0.8% following spinal surgery (47); in the reported cases, the leading causes of rupture of the dura mater before SDH were intravertebral anesthesia (2, 23–25, 32, 40, 43–45), and lumbar surgery (26–31, 34, 35, 37, 41, 42). Other causes of dura rupture include myelography and epidural steroid injection (36, 39, 41). The most common surgery procedures were laminectomy and microdiscectomy in the patients with SDH following spinal surgery (27–30, 34, 35, 37, 42). Moreover, most patients who received intravertebral anesthesia are pregnant women. This result is consistent with previous research showing that pregnancy increases the risk of rupture of the bridging veins following an abrupt pressure variation during labor and delivery (5, 48). According to the literature, other factors that increase the risk of ruptures of the dura mater and the development of ICPR are multiple dural punctures and the sharpness and size of the needle, and cerebral atrophy (34, 44, 49–51). We also found that although dural tears were present in most cases, data relevant to CSF leakage or ICPR (MRI, sign) were primarily unavailable, similar to that in the cases of the current study.

**Table 1:** Cases of secondary ICPR-related SDH.

**Author**	**Case**	**Cause of ICPR**	**SDH time/d**	**Location of SDH**	**Symptoms**	**The treatment of ICPR**	**The treatment of SDH**	**Outcome**
Bjärnhall et al. (23)	71/M	Spinal anesthesia	5	Right SDH	Headaches, nausea and vomiting	/	The conservative treatment	Operation after invalidation in conservative treatment
Bisinotto et al. (24)	48/F	Spinal anesthesia	2	Left SDH	Headaches and vomiting	/	The conservative treatment, analgesics, dexamethasone and hydantoin	Operation after invalidation in conservative treatment
Zeidan et al. (2)	39/F	Spinal anesthesia	30	Right SDH	Headaches, hemiparesis	/	The conservative treatment	CR
Vien et al. (25)	27/F	Epidural anesthesia	3	Right SDH	Headaches, bradycardia and vomiting.	Epidural blood patch	The conservative treatment	CR
Bhimani et al. (26)	10/M	Costoplasty, ect.	5	Right SDH	Drowsiness and convulsions	Repair of lumbar dura	The conservative treatment	CR
Beier et al. (27)	39/F	Microdiscectomy	28	Right SDH	Headaches, nausea	Repair of lumbar dura	The conservative treatment	CR
Watanabe et al. (28)	25/M	Laminectomy	16	Right SDH	Headaches, motor deficit, and dysphasia	/	The conservative treatment with bed rest and intracranial pressure reduction agents	CR
Martínez-lage et al. (29)	6/M	Laminectomy	6	Left SDH	Headaches, syncope, drowsiness and vomiting	/	The conservative treatment with bed rest, intravenous hydration, and dexamethasone	CR
Burkhard et al. (30)	71/M	Diskectomy	21	Bilateral SDH	Headaches, nausea, vomiting, dizziness, tinnitus, and syncope.	/	The conservative treatment with dexamethasone	CR
Magro et al. (31)	41/F	Implantation of intrathecal drug delivery system	42	Bilateral SDH	Headaches, vomiting, and coma	/	The hematoma evacuation	CR
Kelsaka et al. (32)	38/M	Spinal anesthesia.	40	Left SDH	Headache	/	The hematoma evacuation	CR
Kim et al., (33)	39/F	Epidural steroid injection	30	Left SDH	Headache	/	The hematoma evacuation with burr-hole drainage	CR
Lu et al. (34)	59/F	Laminectomy	11	Right SDH	Drowsiness, headaches, nausea, vomiting	/	The hematoma evacuation with burr-hole drainage	CR
Maugeri et al. (35)	57/F	Laminectomy	30	Left SDH	Headaches, hemiparesis	/	The hematoma evacuation with burr-hole drainage	CR
Vos et al. (36)	54/M	Cervical myelography	44	Left SDH	Headaches, dizziness, nausea and vomiting.	Epidural blood patch	The hematoma evacuation with burr-hole drainage	CR
Işik et al. (37)	81/M	Laminectomy	50	Bilateral SDH	Headaches, dysphasia, and facial palsy	Repair of lumbar dura	The hematoma evacuation with burr-hole drainage	CR
Chiravuri et al. (38)	49/M	Placement of spinal cord stimulator	1	Right SDH	Headaches nausea, vomiting, and altered mental status	/	The hematoma evacuation with craniotomy	CR
Suess et al. (39)	57/M	Lumbar myelography	7	Left SDH	Headaches and dizziness	/	The hematoma evacuation with craniotomy	CR
Dawley et al. (40)	26/F	Spinal anesthesia.	1	Right SDH	Headaches nausea, vomiting, altered mental status,	/	The hematoma evacuation with craniotomy	CR
Rosario and Rajan (41)	62/M	Placement and removal of lumbar drain	8	Left SDH	Drowsiness, motor deficit	Epidural blood patch	The hematoma evacuation with craniotomy	CR
Oktay et al. (42)	38/F	Microdiscectomy	21	Right SDH	Headache	Repair of lumbar dura	The hematoma evacuation with craniotomy	CR
Gioia et al. (43)	40/F	Spinal anesthesia	1.5	Left SDH	Headaches and coma	/	The hematoma evacuation with craniotomy	Died
Machurot et al. (44)	53/F	Spinal anesthesia	2	Right SDH	Coma	/	The hematoma evacuation with craniotomy	PR
Ramos-aparici et al. (45)	31/F	Spinal anesthesia	2	Right SDH	Headaches, visual disturbance, altered mental status and hemiparesis	/	The hematoma evacuation with craniotomy	PR
This report	54/M	Cupping therapy	1	Right SDH	Headaches and motor deficit	/	The conservative treatment with ATO and DEX	CR
This report	48/F	Cupping therapy	1	Bilateral SDH	Headaches, nausea, and vomiting	/	The conservative treatment with ATO and DEX	CR
This report	44/M	Cupping therapy	1	Bilateral SDH	Headaches, vomiting and dizziness	/	The conservative treatment with ATO and DEX	Operation after invalidation in conservative treatment

*SDH, Subdural Hematoma; ICPR, Intracranial Pressure Reduction; M, Male; F, Female; ATO, Atorvastatin; DEX, Dexamethasone; CR, Complete Recovery; PR, Partial Recovery*.

Management of these patients mainly includes treatment of the CSF leakage and handling of ICPR. An epidural blood patch is a preferred therapy for dural tear complications, and it comprises injecting autologous blood into the lumbar epidural space. Repair of the lumbar dura is also applied to treat diagnosed dural tears, usually detected during or after spinal cord surgery. They could theoretically be an alternative therapeutic measure in the case of ICPR if diagnosed early (2). However, it is the second-line treatment in everyday practice when conservative treatment fails (17). In the reported cases, ICPR remained untreated in most patients, while few other patients were treated with an epidural blood patch and repair of the lumbar dura (26, 27, 36, 37, 41, 42).

As for SDH, in general, surgical intervention is suggested if the thickness of the hematoma exceeds 10 mm, a midline shift of >5 mm, or neurological deterioration (52). Conservative treatments, including bed rest, administration of ICPR agents, analgesics, DEX, and phenytoin, have been applied to treat patients with mild-symptom ICPR-related SDH. Most patients who underwent surgery had a good prognosis, but one patient was reported to have died at the end of the surgery (43). As for conservative treatment, most patients have completely recovered, but two patients were reported to have deteriorated and subsequently underwent surgery (23, 24). As far as we know, Atorvastatin has never been reported to treat such patients with SDH. We have previously demonstrated that Atorvastatin may be a safe and efficacious non-surgical alternative for treating patients with CSDH (53) and that Atorvastatin and DEX combination treatment is more effective than Atorvastatin-alone in eliminating CSDH (17). Here, our cases indicate that Atorvastatin combined with DEX may be an effective treatment for ICPR-related SDH. However, as one of the patients showed a poor response to this treatment, it is necessary to explore the reasons behind this further.

## Conclusions

Despite the popularity of cupping therapy, its side effects and dangers are rarely mentioned. We reported three cases of SDH that were possibly derived or aggravated by ICPR caused by substandard cupping therapy. From our experience, technicians should fully assess a patient's condition and medical history, and ensure not to place cups at ONJ to avoid injurious results or deterioration of an existing SDH. Moreover, the pathophysiology by which cupping causes SDH needs further investigations.

## Data availability statement

The original contributions presented in the study are included in the article/supplementary material, further inquiries can be directed to the corresponding author/s.

## Ethics statement

The studies involving human participants were reviewed and approved by the Ethical Committee of Tianjin Medical University General Hospital. Written informed consent was obtained from the individual(s) and/or minor(s)' legal guardian/next of kin for the publication of any potentially identifiable images or data included in this article.

## Author contributions

TX is responsible for the writing, data collection, drawing, and reviewing, while RJ is responsible for the review, direction, and financial support. Others are responsible for data collection. All authors contributed to the article and approved the submitted version.

## Funding

The staff received salary support from the General Hospital of Tianjin Medical University and the Neurological Institute of Tianjin. This study was supported by the National Natural Science Foundation of China (*via* grant no.82001323 to CG, grant no. 82071390 to RJ), the Tianjin Research Program of Application Foundation, and Advanced Technology (*via* grant no. 19YFZCSY00650 to RJ) and the Clinical Study of Tianjin Medical University (2017kylc007 to RJ).

## Conflict of interest

The authors declare that the research was conducted in the absence of any commercial or financial relationships that could be construed as a potential conflict of interest.

## Publisher's note

All claims expressed in this article are solely those of the authors and do not necessarily represent those of their affiliated organizations, or those of the publisher, the editors and the reviewers. Any product that may be evaluated in this article, or claim that may be made by its manufacturer, is not guaranteed or endorsed by the publisher.
